# Modeling the spatial-spectral characteristics of plants for nutrient status identification using hyperspectral data and deep learning methods

**DOI:** 10.3389/fpls.2023.1209500

**Published:** 2023-10-16

**Authors:** Frank Gyan Okyere, Daniel Cudjoe, Pouria Sadeghi-Tehran, Nicolas Virlet, Andrew B. Riche, March Castle, Latifa Greche, Daniel Simms, Manal Mhada, Fady Mohareb, Malcolm John Hawkesford

**Affiliations:** ^1^ Sustainable Soils and Crops, Rothamsted Research, Harpenden, United Kingdom; ^2^ School of Water, Energy and Environment, Cranfield University, Cranfield, United Kingdom; ^3^ AgroBioSciences Department, University of Mohammed VI Polytechnic, Ben Guerir, Morocco

**Keywords:** convolution neural network, hyperspectral imaging, plant nutrition, machine learning, spectral curves

## Abstract

Sustainable fertilizer management in precision agriculture is essential for both economic and environmental reasons. To effectively manage fertilizer input, various methods are employed to monitor and track plant nutrient status. One such method is hyperspectral imaging, which has been on the rise in recent times. It is a remote sensing tool used to monitor plant physiological changes in response to environmental conditions and nutrient availability. However, conventional hyperspectral processing mainly focuses on either the spectral or spatial information of plants. This study aims to develop a hybrid convolution neural network (CNN) capable of simultaneously extracting spatial and spectral information from quinoa and cowpea plants to identify their nutrient status at different growth stages. To achieve this, a nutrient experiment with four treatments (high and low levels of nitrogen and phosphorus) was conducted in a glasshouse. A hybrid CNN model comprising a 3D CNN (extracts joint spectral-spatial information) and a 2D CNN (for abstract spatial information extraction) was proposed. Three pre-processing techniques, including second-order derivative, standard normal variate, and linear discriminant analysis, were applied to selected regions of interest within the plant spectral hypercube. Together with the raw data, these datasets were used as inputs to train the proposed model. This was done to assess the impact of different pre-processing techniques on hyperspectral-based nutrient phenotyping. The performance of the proposed model was compared with a 3D CNN, a 2D CNN, and a Hybrid Spectral Network (HybridSN) model. Effective wavebands were selected from the best-performing dataset using a greedy stepwise-based correlation feature selection (CFS) technique. The selected wavebands were then used to retrain the models to identify the nutrient status at five selected plant growth stages. From the results, the proposed hybrid model achieved a classification accuracy of over 94% on the test dataset, demonstrating its potential for identifying nitrogen and phosphorus status in cowpea and quinoa at different growth stages.

## 1 Introduction

Nitrogen (N) and phosphorus (P) are two essential macronutrients that play a significant role in the normal functioning and growth of plants. They are involved in vital plant metabolic processes, such as cell division, protein formation, and photosynthesis (Siedliska et al., 2021). Plants with adequate nitrogen nutrition display green leaves, whilst nitrogen deficiency manifests as chlorosis, starting from light green and progressing to yellow and eventually brown. Phosphorus deficiency inhibits shoot growth and shows decolorized leaves, transitioning from pale green to yellow in severely affected regions (McCauley et al., 2011). When plants are insufficient in nutrients, fertilizer application is required to enrich the soil. However, the application of nitrogen and phosphorus fertilizer often relies on farmer’s experience and intuition, which may result in over- or under-application. This practice can lead to soil quality degradation, crop yield reduction, environmental pollution, and loss of biodiversity (Gao et al., 2021). Hence, accurate estimation and tracking of plant N and P status is essential to promote good agronomic practice and effective fertilizer management.

The tracking of plants’ N and P status traditionally involves visual inspection or laboratory-based chemical analysis, which can be destructive, labor intensive, and prone to error. To indirectly measure nutrient status, breeders and researchers use contact and remote sensing tools. One such tool is the SPAD (Soil Plant Analysis Development) meter, which estimates nitrogen content by measuring the light transmittance of the red (650 nm) and infrared (940nm) wavelengths through plant leaves (Uddling et al., 2007). While this technique is simple and fast, it has limitations. As a leaf contact instrument, it only captures a small area of contact (2 x 3 mm), which may not provide an accurate representation of the spatial variation of nitrogen in plants. Additionally, when these tools are used on a large scale, users may introduce errors and obtain false measurement (Hassanijalilian et al., 2020). To mitigate some of these challenges, image-based non-invasive phenotyping techniques offer a potential solution.

Image-based techniques have proven valuable in plant phenotyping, allowing for the measurement of various plant phenotypic properties such as biomass, leaf area, and plant height (Hairuddin et al., 2011). These techniques utilize images acquired from digital RGB cameras, hyperspectral and multispectral sensors, and 3D laser scanners to extract non-invasive features, trends, and patterns that demonstrate the dynamics of phenotyping traits in response to physiological and chemical changes in plants. Among these imaging sensors, digital cameras are the most widely used for plant phenotyping due to their low cost, portability, and high spatial resolution (Sadeghi-Tehran et al., 2017). However, their spectral limitations, capturing only broad spectral bands of red, green, and blue, present a major challenge. To overcome this challenge, hyperspectral imaging (HSI) has emerged as a promising technique. By combining imaging and spectroscopy, HSI allows for the acquisition of spectral and temporal information from plants, useful for estimating plant physiological parameters (Mishra et al., 2017). HSI samples the reflective areas of the electromagnetic spectrum spanning from the visible regions (400-700 nm) to the short-wave infrared regions (1100-2500 nm). This approach has been successfully applied in non-destructive phenotyping of plant leaf area index (Zhang et al., 2021), plant biomass (Ma et al., 2020), plant nutrient estimation (Ye et al., 2020), and detection of diseases and fungal infections (Siedliska et al., 2018).

HSI data are presented in a hypercube format, with the first two dimensions providing spatial information and the third dimension representing spectral information. Extracting relevant information from these hypercubes requires advanced pattern recognition algorithms, such as machine learning (ML) (Singh et al., 2016). ML algorithms learn patterns and trends from data without relying on explicit programming. In a study by Yamashita et al. (2020), ML regression models were employed to analyze differences in spectral reflectance and estimate N and chlorophyll contents in tea plants. A data-based sensitivity algorithm was applied to select the most informative spectral bands capable of estimating N and chlorophyll contents in tea leaves. The results showed that the integration of spectral data with machine learning models is a promising technique for accurate plant nutrient estimation. To further improve the application of ML models to HSI, researchers are currently exploring deep learning methods.

Deep neural networks, particularly convolutional neural networks (CNNs), have emerged as state-of-the-art machine learning techniques capable of detecting, classifying, and predicting plant phenotypes. In the context of hyperspectral imaging (HSI), CNNs have been successfully combined with HSI for various applications such as crop and weed classification (Dyrmann et al., 2016), plant seedlings classification (Nkemelu et al., 2018), and plant disease and pest detection (Sladojevic et al., 2016; Fuentes et al., 2017). Most traditional deep learning methods employed for HSI extract either spectral or spatial information separately (Hong and He, 2020). For instance, 2D-CNN models focus on spatial information, whilst 1D-CNN models primarily utilize spectral information from HSI. In Roy et al. (2020) and Mu et al. (2021), combining both the spatial (2D) and spectral information (1D) information gave better results than using only the spectral or spatial information. However, since these two are extracted separately, there is difficulty in fully utilizing all information from a hypercube simultaneously. To address this limitation and effectively utilize the structural traits of the HSI data, researchers have introduced 3D-CNN methods (Mirzaei et al., 2019; Pi et al., 2021). Indeed, 3D-CNN modeling naturally suits the spatial–spectral information of a hypercube and presents a promising approach for modeling various scenarios. For example, Al-Sarayreh et al. (2020) proposed a novel 3D-CNN model to extract combined features to classify meat using HSI imaging, while Jung et al. (2022) developed a 3D-CNN classification model for diagnosing gray mold disease in strawberries. In both studies, the models employed 3D convolutions, filters, and kernels to extract relevant spatial and spectral information from the hypercube. However, homogeneous 3D models pose difficulties in optimization due to increased parameters, resulting in lengthy computational time, over-fitting, and gradient disappearance. These challenges are attributed to the increased complexity of the models. To further understand CNN modeling using HSI data, readers are referred to Kumar et al. (2020); Paoletti et al. (2019); Huang et al. (2022); Wang et al. (2021) and Jia et al. (2021) for more details. To mitigate some of these challenges, researchers have proposed the use of hybrid CNN models, which fuse two-unit deep learning blocks together to simultaneously extract both spectral and spatial information from HSI data. For instance, Mohan and Venkatesan (2020) proposed a multiscale hybrid CNN model for hyperspectral image classification. This model extracts spatial–spectral features from different window sizes using a 3D-CNN block. The output from the 3D CNN block is concatenated and fed into a 2D CNN block for abstract spatial feature extraction. The performance of the proposed model showed satisfactory performance when compared to state-of-the-art CNN-based models. Although this method works effectively in simultaneously extracting spectral and spatial features from hypercubes, there is a considerably high number of trainable parameters, which affects the processing time.

In this study, we developed a hybrid model capable of extracting spatial and spectral information simultaneously from a hypercube with reduced computational complexities and time. The proposed model is to identify plant nutrient status (N and P) at different growth stages. Specifically, hypercube regions of interest (patches) measuring 15x15-pixels were extracted from four nutrient treatments. Different spectral transformation techniques were applied to obtain four distinct datasets. The resulting datasets were each used to train the proposed model to classify plants based on their nutrient status. Furthermore, a waveband selection method was applied to the dataset produced by the best performing dataset. The selected wavelengths were used as inputs to retrain the proposed model to identify plant nutrient status at different growth stages.

## 2 Materials and methods

**Figure 1:**
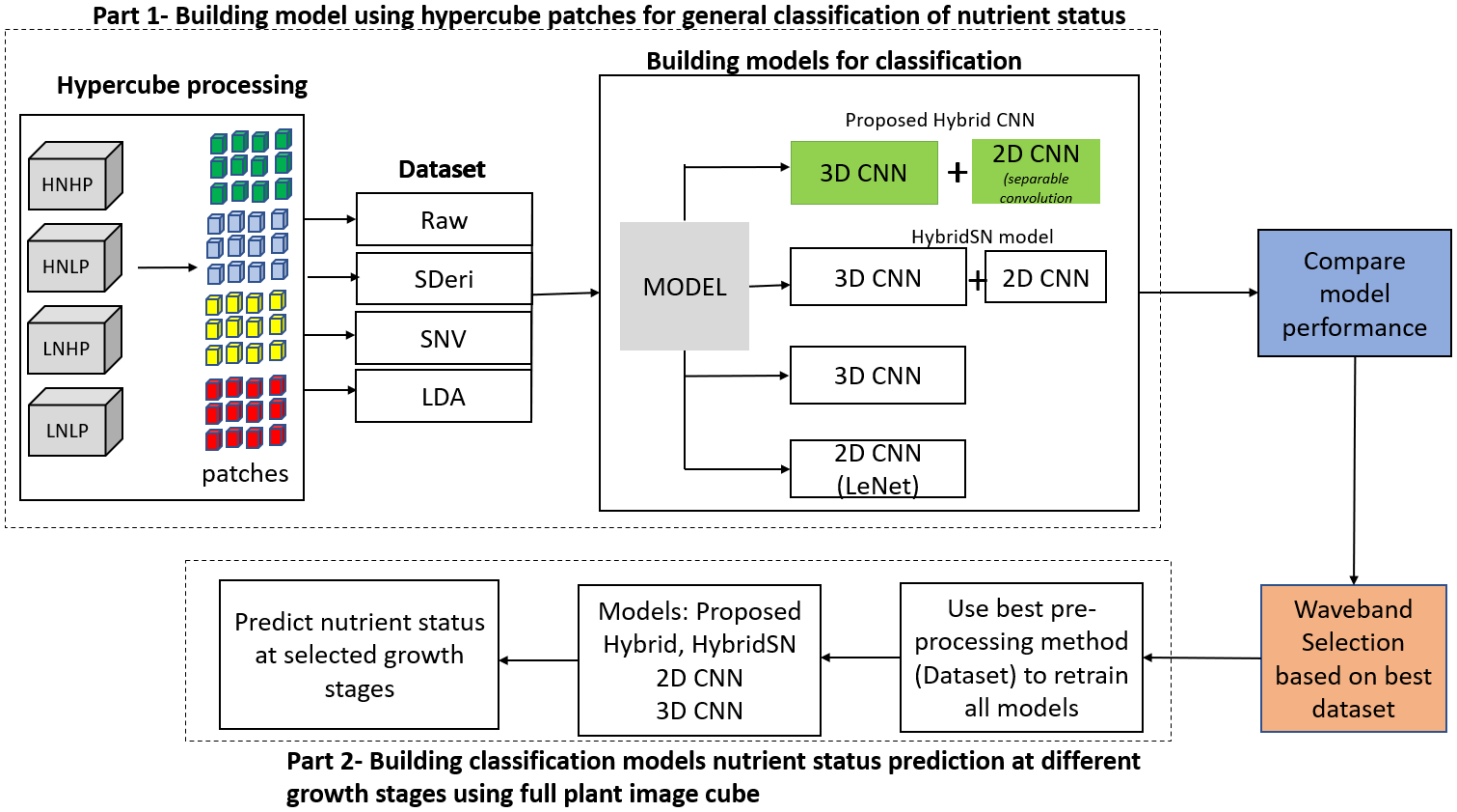
Workflow of the experiment.

**Figure 1** is the workflow of this study. It involves the generation of the spectral library of hypercube patches to build four different datasets. These datasets were used to train four models to classify plant nutrient status. The most informative dataset was then used to retrain the models based on the selected wavebands from the full plant data (not patches) to classify plants at selected growth stages.

### 2.1 Plant material and treatments

*Vigna unguiculata* (cowpea) and *Chenopodium quinoa* (quinoa) were cultivated in a Plant Growth Facility https://www.cranfield.ac.uk/facilities/plant-growth-facility) at Cranfield University (Cranfield, UK). The plants were grown in pots filled with compost with reconstituted nutrients. The compost was first washed with deionized water following the works of Masters-Clark et al. (2020) with some modifications. This was done by flooding one-part compost with five-part deionized water. A double-headed 0.5 µm sieve was used to drain off the soluble solution. The steps were repeated five times, ensuring the plant nutrients were depleted, and the washed compost was dried in an oven at 70°C for 72 hours. Based on the Letcombe nutrition solution (Masters-Clark et al., 2020), the required treatments were prepared as: high nitrogen high phosphorus (HNHP), high nitrogen low phosphorus (HNLP), low nitrogen high phosphorus (LNHP) and low nitrogen low phosphorus (LNLP), with five replications each for the treatments. The treatments had concentrations of 49.1 mM and 14.6 mM N for HN and LN and 13.4 mM and 3.3 mM P for HP and LP, respectively. By visual inspection, the different treatments resulted in changes in the leaf pigments, resulting in variations in the spectral characteristics during plant development. For both species, the leaves were green when the nitrogen and phosphorus levels were adequate, whilst deficiency in nitrogen was characterized by chlorosis progressing from light green to yellow to brown. Phosphorus deficiency inhibited shoot growth and decolorized leaves from a blue-green color to pale green/yellow in severely affected regions (McCauley et al., 2011).

### 2.2 Hyperspectral imaging system and data collection

Spectral images were collected 3.0 m above the canopy level using a Lemnatec Scanalyzer installed in the glasshouse. The Scanalyzer has a push broom hyperspectral camera (hyperspec^®^ inspectorTM Headwall Photonic) that operates within the 390 – 2500 nm region covering the visible–near-infrared (VNIR) and short-wave infrared (SWIR) regions. The sensor collects data with a 0.7 nm step (at the VNIR region) and 6 nm step (within the SWIR region) and an FWHM (full width at half maximum) image slit of 2.5 nm. This results in a hypercube of 1015 spectral bands for the VNIR data and 275 spectral bands for the SWIR data, with a dynamic range of 16 bits. Data collection was performed twice per week throughout the full development of the plants. Five different growth stages of cowpea and quinoa, hereafter referred as growth stages I, II, III, IV, and V, were considered for this study. They represent the 19, 29, 36, 50, and 69 DAT (days after transplanting) and 13, 30, 51, 60, and 72 DAT for the cowpea and quinoa plants, respectively. **Supplementary Table 1** is the description of the selected growth stages based on the BBCH system for coding the phenological growth stages of plants (Meier et al., 2009).

### 2.3 Pre-processing of raw spectral data

Prior to any analysis, the raw data were pre-processed to normalize the spectral data from ambient illumination and to reduce noise and other artefacts that were produced during scanning. The pre-processing steps comprised: (i) radiometric calibration to remove illumination system effect, (ii) spectral down-sampling to remove redundant wavelengths, and (iii) noise reduction.

*Radiometric calibration:* Hyperspectral image acquisition suffers from radiometric errors caused by illumination from varying light exposure. Radiometric correction is essential to reduce the variable illumination effect and the influence of dark current on the spectral data. During scanning, a white panel (Zenith Lite™ Ultralight Targets 95%R, Sphereoptics^®^) was imaged as the white reference data. Dark reference data with approximately 0% reflectance were collected in the night without any light source by completely covering the camera lens with an opaque cap (Zhu et al., 2013). The calibrated data were calculated using equation (1):


(1) $$\text{Ic}=\frac{{\text{I}}_{\text{raw}-{\text{ R}}_{\text{dark}}}}{{\text{R}}_{\text{white }–{\text{ R}}_{\text{dark}}}}$$

Where Ic is the calibrated data, I_raw_ is the raw spectral data, R_dark_ is the dark reference, and R_white_ is the white reference.

*Down-sampling*: Although the hyperspectral data contain essential plant information, the huge dataset poses computational challenges. Down-sampling helps to reduce these computational complexities and the noise generated during scanning (Sadeghi-Tehran et al., 2021). Moreover, down-sampling is conducted to map the spectral resolution to its reference target. In this study, an averaging window with a 2 nm spectral width was used to down-sample the spectral data.

*Spectra smoothing and denoising*: This is a common pre-processing practice that involves some numerical operations on the raw spectral data to reduce spectral noise levels. This eliminates spikes and smooths spectral curves whiles isolating essential features, which may be obscured by the presence of noise across the different wavelengths. During spectral smoothing and denoising, the original shape and features of the spectra are normally preserved. In our study, we applied the Savitzky–Golay filter (a commonly used low-pass filter), an effective and computationally fast filter, to smooth the spectra. For each data point in the spectrum, the filter selects an odd-sized window of spectral points and fits a least-square regression with a polynomial of higher order. During this operation, the data points in question are eventually replaced with the corresponding values of the fitted polynomial. We used a window of size 13- and second-degree polynomial as the optimal parameters. It should be noted that a small window size leads to the emergence of large artefacts in the smoothed spectra, whilst the larger the window size, the smaller the distinction between full and moving window processing (Rinnan et al., 2009).

*Segmentation:* After pre-processing, the HSI data were segmented using a selected spectral ratio combined with Otsu thresholding. To segment the VNIR dataset, 705 nm and 750 nm wavelengths were extracted to create a red-edge normalized difference vegetation index (RENDVI) image, which was then combined with automatic Otsu thresholding for segmentation. The RENDVI generally differentiates plant vegetation from non-vegetation regions. Hence, the combination with Otsu thresholding creates a binary image where the plant images are labeled as one and all other items labeled as zeros. The SWIR dataset segmentation used a similar approach, where the NDVI image was derived from wavelengths 1375 nm and 1141 nm, as proposed by Williams et al. (2017).

### 2.4 Data transformation

Although HSI contains important information that relates objects to its absorptance and reflectance, the measured spectra are subject to spurious signals such as multiplicative and additive effects. Hence, further pre-processing steps are applied to minimize the effect of undesirable occurrences on spectral measurement such as artifacts, particle size effects, and light scattering (Amigo, 2010). In this study, the effects of different pre-processing steps on the performance of classification models were studied by transforming the HSI data using standard normal variate (SNV), second derivative (SDeri), and linear discriminant analysis (LDA). In addition to the raw reflectance spectra, three datasets were generated from the SNV, SDeri, and LDA, respectively, to train the models.

#### 2.4.1 Standard normal variate

SNV is a pre-processing technique aimed at limiting the multiplicative effect of scattering and particle size. It accounts for the variation in baseline shift and curvilinearity in the reflectance spectra and reduces the difference in the global intensities of the reflectance spectra (Luo et al., 2019). SNV transformation was performed on each individual spectrum, requiring no reference spectrum. The raw spectra were transformed using the SNV technique by finding the mean center of each spectrum and dividing by its standard deviation (Barnes et al., 1989), as shown in equation 2:


(2) $$x_{i,j}^{\text{SNV}}=\sqrt{\frac{\sum_{j}^{y}x_{i,j-\;}{\bar{x}}_{i}}{y-1}}$$

Where *x_i,j_* ^SNV^ is the element of the transformed spectrum, *x_i,j_* is the corresponding original element of the spectrum *i* at measured at wavelength *j*, ${\bar{x}}_{i}$ is the mean reflectance of the uncorrected spectra *i*, and *p* is the number of variables or wavelengths in the spectrum.

It should be noted that SNV is performed independently for each pixel (has zero mean and variance equal to one). Hence, it gives an advantage over averaging methods such as MSC (Multiple-Scattering Correction), where the presence of non-plants could influence the averaging process.

#### 2.4.2 Spectral derivatives

The spectral derivative aims to normalize the spectral differences between two continuous narrow bands and remove or suppress image artefacts that result from non-uniform illumination (Pandey et al., 2017). It increases the spectral resolution of overlapping peaks whilst accounting for the baseline correction of reflectance spectra (Perez-Sanz et al., 2017). According to Cloutis et al. (1996), the spectral derivative is sensitive to the signal-to-noise ratio (SNR), such that the higher the spectral derivative, the higher the noise and vice versa. From literature, the first and second derivatives are more effective in managing different spectral disparities and improving data modeling. In this study, we employed the second derivative (SDeri) using the Savitzky–Golay smoothing and polynomial derivative package in Python. **Supplementary Figure 1** shows the hypercube of cowpea with examples of the raw, SNV, and SDeri spectral curves.

#### 2.4.3 Linear discriminant analysis

The high spatial and spectral resolution of the data in this study posed a computational challenge to the analysis of the hypercube. Hence, it was desirable to apply a dimensionality technique to reduce the depth of the hypercube while maintaining the core informative features of the data. To do so, the hypercube defined as (*w* x *h* x λ) was rearranged into a 2-d spectral matrix (M x λ), where M is the product of w and h. A linear discriminant analysis (LDA) was applied to find the linear combination of spectral features that characterizes the different treatments. LDA is a probabilistic generalization technique that aims at projecting features in higher dimensions to lower dimensions for solving classification and regression problems (Xia et al., 2019). It reduces the size of a dataset while retaining the relevant information that discriminates the different classes. Unlike principal component analysis (PCA), which takes in only the spectral features and its variances irrespective of their classes, LDA makes use of the different class labels to maximize the differences in the classes. **Supplementary Figure 2** is a scatter plot of the first and second discriminants using LDA for cowpea and quinoa. Comparing **Supplementary Figures 2**, **3** for LDA and PCA, respectively, it is observed that LDA performed better in separating the four classes. In the PCA, the classes were not as clearly separated, even though together the first two principal components contained over 90% of the class information.

### 2.5 Dataset for model training

#### 2.5.1 Generating hypercube patches

The original HSI hypercube obtained from the Scanalyzer contained 1397 spectral dimensions (1015 and 275 for VNIR and SWIR, respectively) for each plant. Down-sampling was applied on both species, reducing the datasets to 223 (155 VNIR and 68 SWIR) spectral dimensions for further analysis. To build the classification model, an automated algorithm was created to extract hypercubes from the four datasets (raw, second derivative, LDA, and SNV data). This was done by building a spectral library of region of interest (patches) from the plant leaves. A patch (**Supplementary Figure 4**) is defined as a square area of size 15 x 15 pixels and has a spectral dimension with λ number of wavelengths. From a hyperspectral image (H ∈ $I^{\;(h\;\times \;w\;\times \;y}$ ), n number of patches (P ∈ $I^{\;\left(m\;\times \;n\;\times \;k\right)}$ ) can be produced depending on the patches size required, where h x w and m x n represent the width × height dimensions of the hyperspectral image and patch hypercube, respectively, while y and k denote the spectral bands for patches and the image respectively. To produce the patches, the hypercube containing the whole plant was divided into four equal segments. A function that utilizes the *patchify library* in python was developed to create patches on each segment, as shown in **Supplementary Figure 4**. Then, 20 non-zero patches from each segment were extracted, producing 80 patches per image and resulting in 2800 patches per treatment. The patches for each species were generated from 35 (7 time points and 5 replications) hyperspectral images per treatment.

#### 2.5.2 Developing the training dataset

Since the hyperspectral data for both species from each treatment were divided into 2800 sample patches, there was a total of 11,200 patches used for the experiment. Before extracting the hypercube patches for training the models, the original hyperspectral data were first divided into a 3:1 train–test dataset. The train dataset was further split into an 80%-20% train–validation dataset. The number of samples of the training, validation, and test set for each model is shown in **Table 1**. To introduce spatial variations into the dataset, the 40% of the train–validation dataset was rotated at different angles between -20° and 20°. All this was done for both species.

**Table 1:** The number of training, validation, and testing samples.

Number of samples (patches)	Number of Treatment	Training set	Validation set	Test set
2800	4	1680	420	700
2800	4	1680	420	700
2800	4	1680	420	700
2800	4	1680	420	700

### 2.6 Developing models for spatial–spectral characterization of HSI data

#### 2.6.1 Hybrid CNN framework hyperspectral classification

In this study, we employed a hybrid 3D-2D CNN method to model the spatial–spectral variations and interclass appearance to improve the power of accurately differentiating the N and P variations in cowpea and quinoa. The next paragraph explains the architecture and operations of the two units of the model.

*3D Convolution Unit*: This block employs a 3D convolution technique where the kernel slides in three dimensions convolving input data in their spatial and spectral dimensions, resulting in a 3D data output. It is made of three 3D convolution layers interspersed with layers of filters ranging from 64, 32, and 16, constituting a (3 x 3x 3), (3 x3 x 5), and (3 x 3 x 7) kernel size, respectively. In addition, the filters and the generated feature maps are all configured in a 3D format. The 3D convolution operation is given by equation (3) (Mohan and Venkatesan, 2020):


(3) $$y_{qrs}=f(\sum_{r}\sum_{i=0}^{h-1}\sum_{j=0}^{w-1}\sum_{l-0}^{s-1}k_{ijl}x_{\left(i+q\right)\left(j+r\right)\left(l+s\right)}+b_{qrs}$$

where *y_qrs_* denotes the extracted feature at position *(q, r, s)* and *k* is the kernel at dimension *ijl*. S is the kernel size in the spectral dimension.

*2D CNN Unit*: In this block, the input is convolved with 2D kernels and filters where the convolution is mathematically obtained by the sum of the dot product between the kernels and the input data. The kernel strides over the entire data to acquire the spatial information. As mentioned by Mohan and Venkatesan (2020), the 2D convolution process can be mathematically expressed as:


(4) $$T_{mn}=\sum_{l}\sum_{i=0}^{h-1}\sum_{j-0}^{w-1}k_{ij}x_{\left(i+m\right)}\left(j+n\right)+bias_{mn}$$

where *T_mn_* is the feature extracted at position *(m, n)* and *k* is the kernel (2D) at dimension *ℎ×w*. This convolution operation is applied on all feature maps *(l)* in the receiver area summing all the values for non-linear activation.

**Figure 2:**
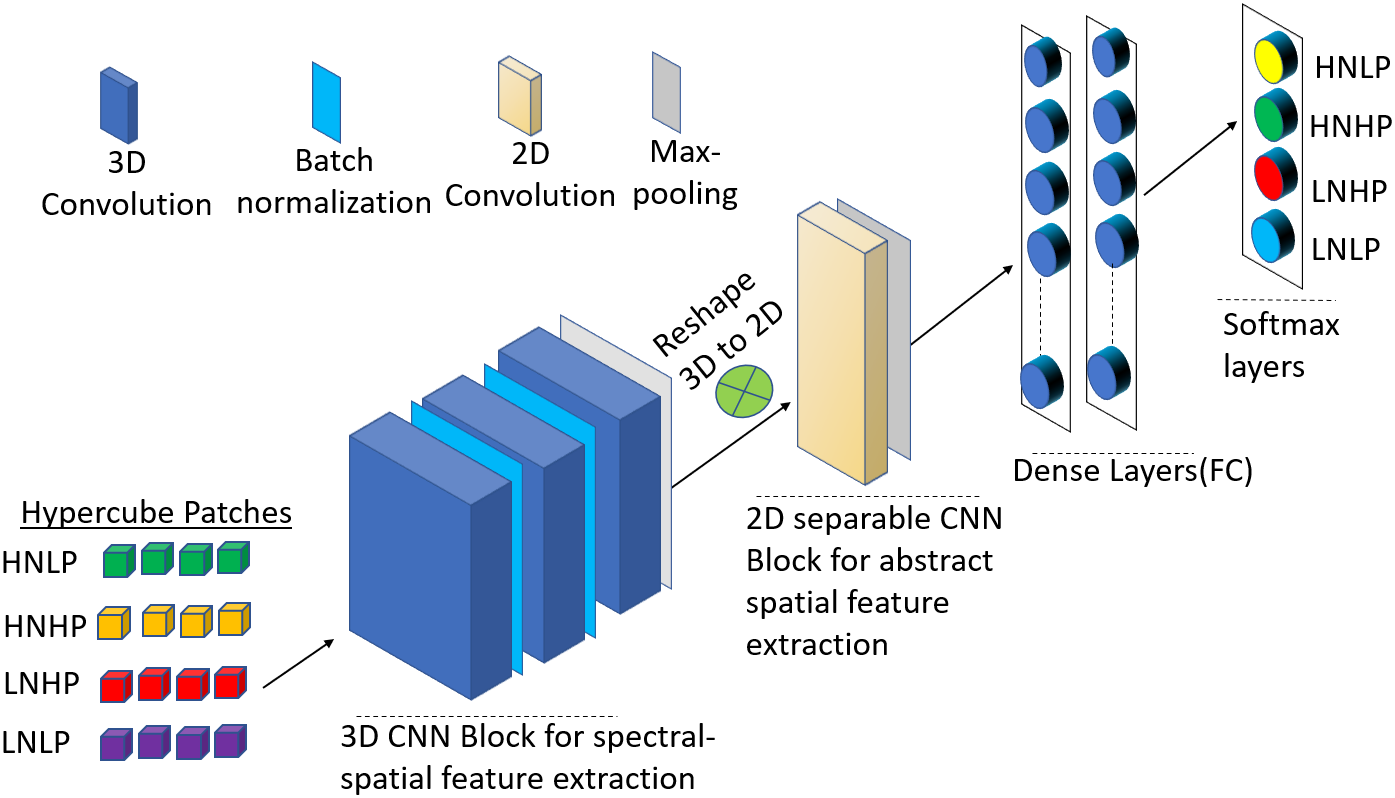
Schematic representation of the proposed 3D-2D-CNN framework with the full-spectrum bands as inputs.

This block has one 2D convolution layer (separable in nature), a max pooling layer, and two fully connected (FC) layers, and SoftMax layers. The separable convolution is a transformation form of conventional 2D convolution in which a single convolution is divided into two or more convolutions to get the same output. There are two steps in separable convolution: spatially and depth-wise separable convolution (Fırat et al., 2022). Spatially separable optimizes the performance of the convolution networks to help preserve the spatial information of the data, while the depth-wise convolution decreases the number of trainable weight parameters while increasing representation efficiency. To prevent the model from overfitting, a 0.5 probability dropout was used on each fully connected layer. The output is fed into a SoftMax layer. **Figure 2** summarizes the hybrid CNN architecture for this study. **Table 2** also shows the output dimension and number of parameters used in each layer for the hybrid model.

**Table 2:** The proposed hybrid-CNN algorithm structure.

Layer (Type)	Output Shape	Number of Parameters
Input_layer1 (input layer)	15, 15, 223, 1	0
Conv3D (Conv3D)	13, 13, 221, 8	224
batch normalization	13, 13, 221, 8	32
Conv3D-1 (Conv3D)	11, 11, 219, 8	1736
batch normalization	11, 11, 219, 8	32
Conv3D-2 (Conv3D)	9, 9, 217, 16	3472
MaxPooling3D	4, 4, 108, 16	0
Reshape 3D-2D	4, 4, 1728	0
separable_conv2d-2 (Separable Conv2d)	2,2,32	70880
MaxPooling2D	1, 1, 32	0
Flatten	32	0
Dense	256	8448
Dropout	256	0
Dense-1 (Dense)	128	32869
Dropout	128	0
Dense-2 (Dense)	4	516

#### 2.6.2 Other models for comparison (3D CNN, 2D CNN, and HybridSN)

The performance of the proposed method was compared to other methods such as hybrid spectral convolution neural network (HybridSN), 3D-CNN, and 2D- CNN models. The HybridSN is a modification of the model proposed by Roy et al. (2020) for HSI classification. It is a 3D-2D CNN joint model that combines the complementary spectral–spatial information from the 3D-CNN component and abstract level information from the 2D CNN model. In this model, the first three layers are homogeneous 3D convolutions, with a 3x3x3 kernel size in the first and third layers and 3x3x5 filter kernels in the second layer. Each layer has 8, 16, and 32 filters, respectively. The output from the last layer of the 3D block is reshaped and fed into the 2D CNN block. This block is made of a classical 2D convolution with a 3 x 3 kernel size and 64 filters. The output is flattened and passed through two dense layers with Dropout. This is followed by a single SoftMax layer corresponding to the output. **Supplementary Table 2** shows the detailed architecture of HybridSN used in this study.

The 3D-CNN model for hyperspectral data modeling is known for extracting spectral and spatial information simultaneously from hyperspectral data. It is especially useful when relevant information is localized in both the spatial and spectral dimension and exhibits good correlations in both domains (Nagasubramanian et al., 2019). The 3D-CNN model used in this study has four 3D convolution layers, with 3D kernel of size 3 x 3 x 3 used for the input of the first convolution layer and three kernels of sizes 3 x 3 x 5 in between the other three layers. The convolution layers are interspersed with max pooling and batch normalization layers between every two convolution layers. It uses the rectified Linear Input (ReLU) activation function for each convolution output (Glorot et al., 2011). There are two fully connected layers that follow the last convolution layer. Dropout with a 0.5 probability was performed after the first max pooling operation. The dropout process was used to prevent overfitting during training. The last fully connected layer was fed to the SoftMax layer for classification. **Supplementary Table 3** is the detailed architecture of 3D CNN for the classification of nutrient status.

The 2D-CNN model is a modified version of LeNet-5 deep learning architecture introduced by LeCun et al. (1998). This model was chosen due to its simple and straightforward architecture. It is made of two convolution layers with two pooling layers interspersed between the convolutions. It also has a flattening layer, two fully connected layers, and a SoftMax layer that classifies the resulting features. The convolution layer is responsible for generating feature maps by sliding the given filters over the input data and recognizing patterns and trends. The first convolution layer has a 3 x3 kernel size, and a stride of one which outputs feature maps of sizes 11 x 11 x 6, while the second convolution layer with a 3 x3 kernel size, and a stride of one takes in 11 x11 x6 input feature maps and outputs 7 x 7x12 feature maps. The pooling layer at each end of the convolution layers is made of filters of size 2 x 2, and a stride of two which down-sampling the feature maps by calculating the average value of the patches of each feature map. The first pooling layer halves the sizes of the feature map from 11x11x 6 to 5 x 5 x 12, while the second pooling layer halves the sizes of the feature map from 11x11x 6 to 5 x 5 x 12. The fifth and sixth layers are fully connected layers with 120 feature maps of sizes 1 x 1 and 140 features maps of sizes 1 x 1, respectively. In addition, ReLU activation function is used in this architecture. **Supplementary Table 4** shows the detailed architecture of the 2D-CNN model (LeNet) used in this study.

The four models were each trained with RAW, SNV, SDeri, and LDA datasets, resulting in four models for each dataset. For the validation models, a stratified 10-fold cross validation with five repeats was conducted. All the models were programmed in Python 3.8 and implemented based on TensorFlow and Karas open-source framework. The operating platform was on a PC with Intel (R) Core (TM) i7-370K U CPU with 3.50 GHz and 16 GB RAM. All the classification algorithms were established using the full spectrum (390–2500 nm), and the same parameters (window size, training sample, testing sample) were set for a fair comparison.

### 2.7 Optimal wavelength selection

Hyperspectral data contain a large amount of information that could be highly redundant and multi-collinear within adjacent wavelengths, resulting in computational complexities during processing and application (Hennessy et al., 2020). Feature mining methods that extract the most relevant and sensitive informative wavelengths from the spectral data are important to reduce multi-collinearity in the data and enhance model robustness. In this work, a correlation-based feature selection (CFS) method was applied to the best group of spectral data. CFS is one of the most popular data engineering methods for selecting a sensitive set of features to build a discriminative model for a specific purpose. It works on the principle that good and informative wavelengths are those that are highly correlated with a particular class but uncorrelated with each other. To use the CFS algorithm, a heuristic search algorithm was applied along with a correlation function (Pearson’s correlation) to assign high scores and select the best subset of features that had high predictive ability of the class labels with poor correlation with each other. A greedy stepwise (GS) search strategy was applied during the application of the CFS algorithms to select the space attributes of the variable subsets.

### 2.8 Evaluation metrics for model performance

To evaluate the performance of the proposed model against the classification models, the overall accuracy (OA), the F- Score, and the kappa coefficient (Kappa) evaluation metrics were considered. OA represents the number of correctly classified samples in the overall test samples, which is given by equation 5. The F-score evaluates the accuracy of the model on the entire dataset and is useful when there is an uneven class distribution. It is given by the expression shown in equation 6. The kappa coefficient is a statistical measure that describes the mutual information between the ground truth map and the predicted classified map. Kappa values ranges between 0 and 1, such that a coefficient of 1 means perfect agreement with predicted class and ground truth and vice versa when the coefficient is 0.


(5) $$\;\text{OA}=\frac{\sum_{y=1}^{x}d_{ii}}{N}$$

where OA is the overall accuracy, N is the number of all samples, x is the number of class labels present in the dataset, and dii is obtained from the diagonal element of the confusion matrix.


(6) $$\text{F}-\text{Score}=2x\frac{\text{Precision X Recall }}{\text{Precision + Recall }}$$

where $\text{Recall}=\frac{\text{TP}}{\text{TP }+\text{ FN}}$ and $\text{Precision}=\frac{TP}{\text{TP + FP}}$ and TP is true positive, FN is false negative, and FP is false positive.


(7) $$\text{K}=\frac{P_{o-\;}P_{c}}{1-\;P_{c}}$$

where P_o_ is the probability of observed agreements and P_c_ is the expected agreement.

## 3 Results

### 3.1 Characteristics of spectral curves for different nutrient treatment

**Figure 3:**
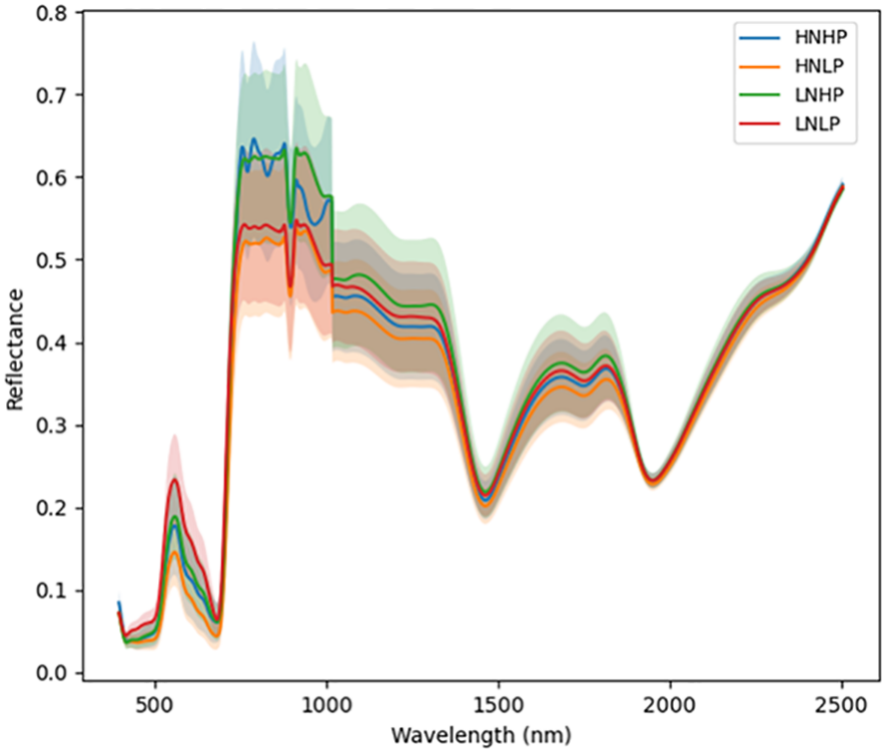
Average reflectance spectra for cowpea under different N and Plevels obtained at growth stage III. Each line represents the averagespectral signature for five plants from each treatment of theexperiment.

**Figure 4:**
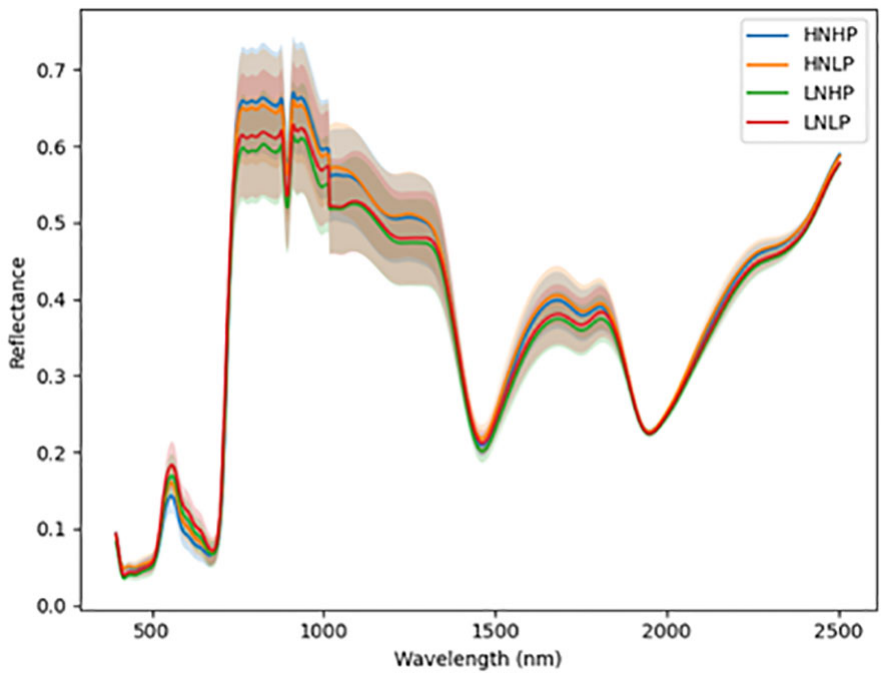
Average reflectance spectra for quinoa under different N and P levels obtained at growth stage III. Each line represents the averagespectral signature for five plants from each treatment of the experiment.

**Figures 3** and **4** represent the average spectral curves for cowpea and quinoa, respectively, at growth stage III. For both species, although the spectral curves are similar in shape, there are visible differences between the four treatments. There is a characteristic peak in the visible spectra observed, especially for wavelengths between 550 nm and 560 nm, for both plant species. This indicates a high amount of chlorophyll absorption in this region (Siedliska et al., 2021), which shows that chlorophyll could be a responsive feature for the dynamics of N and P in both plants. The region between 600 nm and 700 nm (red band) shows a clear and continuous distinction between some variants of the experiment for both species. The low-nitrogen treatments exhibited higher reflectance in contrast to the high-nitrogen treatments, which had low reflectance in this region. HNLP had the lowest reflectance, while LNLP had the highest reflectance in cowpea. Similarly, LNLP and HNHP had the highest and lowest reflectance in the red-edge region for quinoa. This agrees with Clevers and Gitelson (2013), who demonstrated rapid change in the reflectance of the plant canopy in the red-edge region in response to high levels of nitrogen. The regions between 750 and 1100 nm (near infrared) exhibited the highest differences between the spectral curves of the two species. The HNHP had the highest reflectance in both species at above 0.62 nm and above 0.64 nm for cowpea and quinoa, respectively. This agrees with Zhai et al. (2013), who showed that reflectance of healthy plants (in this case, HNHP plants) in the NIR region has a strong correlation with the chemical and cellular architecture of plants and exhibits high reflectance. In the SWIR region, there was a characteristic drop in reflectance from 1250 to 1500 nm and a gradual rise and fall in reflectance across the spectra from 1500 to 2000 nm. Moreover, there was comparably low reflectance in the two - related treatments at 1450 nm, which agrees with Siedliska et al. (2021), who suggest that this region has a quantitative relationship between light reflectance and P treatments. From wavelengths 2000 nm to 2500 nm, there was an observable increase in reflectance for both species.

### 3.2 Model performance based on different pre-processing methods

**Figure 5:**
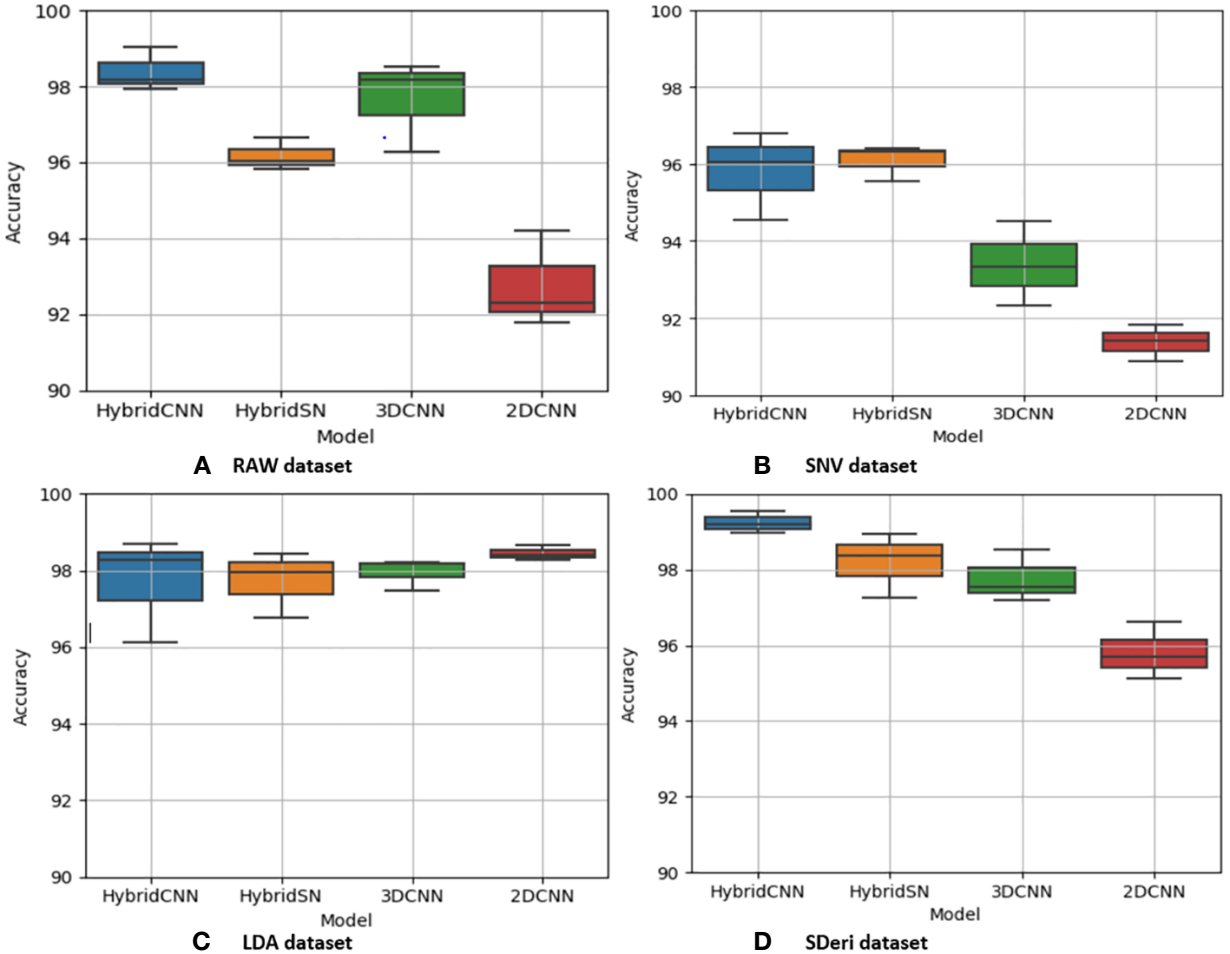
Box and whisker plot of classification accuracy scores for cowpea for the four models: Hybrid-CNN, 3DCNN, 2D CNN, and HybridSN. **(A)** Raw dataset, **(B)** SNV dataset, **(C)** LDA dataset, **(D)** Hybrid CNN dataset.

**Figure 6:**
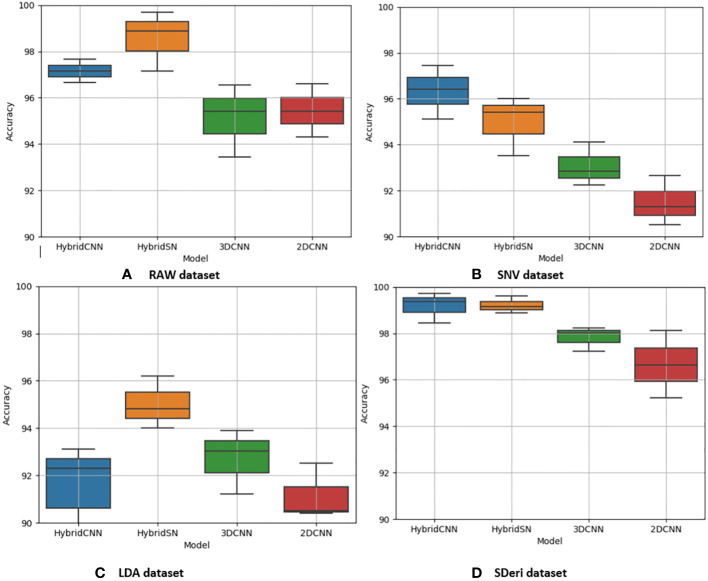
Box and whisker plot of classification accuracy scores for quinoa for the four algorithms: Hybrid-CNN, 3DCNN, 2D CNN, and HybridSN. **(A)** Raw dataset, **(B)** SNV dataset, **(C)** LDA dataset, **(D)** Hybrid CNN datas.

**Figures 5** and **6** are box and whisker plots summarizing the accuracy distribution scores for the different models of the two species. **Tables 3**, **4** summarize the performance of each model based on the evaluation metrics used in this study. From **Figure 5**, the pre-processing methods had an effect on the performance of the different models. SDeri datasets had higher accuracies in all four models, achieving the highest OA (99.24%), kappa (98.64%), and F-score (99.19%) for cowpea and 99.18% OA, 98.85% kappa, and 98.76% F-score for quinoa. The high OA and F-score show the good generalization capacity of the models in classifying the nutrient status. In contrast, the RAW dataset produced the lowest performance for all the models, with the hybrid CNN model achieving the highest performance for cowpea (98.39%, 98.37%, and 97.81% OA, F-score, and kappa coefficient, respectively). In quinoa, the HybridSN model achieved the best performance when trained on the raw dataset with 98.57%, 98.43% and 97.76% OA, F-score, and kappa coefficient, respectively. On the contrary, the 2DCNN model (trained with raw dataset) had the lowest performance with 92.77% OA for cowpea. Furthermore, the 2DCNN model had the lowest performance (91.15% OA) when trained with the LDA dataset for quinoa. There was a 3.58% and 1.57% reduction in accuracy performances when the hybrid model was trained with the SNV and LDA datasets, respectively, for cowpea as compared to the performance when trained with the SDeri dataset. Similarly, the proposed model decreased in performance when trained with the SNV and LDA datasets, achieving 96.32% and 91.45% OA scores, respectively, for quinoa. This suggests that the SDeri dataset had important learnable wavelengths to discriminate the treatments for both species. Although the LDA dataset had reduced training samples, all the LDA-based models displayed lower performance compared to the SDeri dataset-trained model’s performance.

**Table 3:** Classification accuracy assessment and computation cost with different models and pre-processing methods for quinoa.

Model	Quinoa				
	Pre-processing datasets				
	Metrics	RAW	SDeri	SNV	LDA
Hybrid-CNN	OA	97.15±0.51	99.18±0.66	96.33±1.15	91.45±2.22
	F-Score	96.85±0.26	98.85±0.26	95.79±2.17	91.46±1.44
	Kappa	96.49±0.44	98.76±0.65	95.88±1.28	90.68±2.13
	Training Time(s)	1021.06±75.44	1017.93±29.98	1033.03±35.25	252.43±12.24
3DCNN	OA	95.14±1.58	97.82±0.531	93.07±0.96	92.71±1.36
	F-Score	95.36±0.49	95.36±0.49	92.51±1.82	91.90±1.29
	Kappa	94.57±2.04	97.05±1.70	91.603±1.68	91.33±2.11
	Training Time(s)	2274.73±14.88	2220.80±51.00	23104.96±42.84	983.16±15.34
2DCNN	OA	95.44± 0.56	96.657±1.445	91.49±1.08	91.15±1.18
	F-Score	94.27±1.811	94.27±1.811	90.84±1.34	90.59±1.00
	Kappa	93.68±1.636	95.23±0.94	90.43±1.03	90.38±2.60
	Training Time(s)	498.51±18.06	456.30±75.32	672.96±55.09	119.06±61.99
HybridSN	OA	98.57±1.30	99.01±0.38	94.98±1.31	95.01±1.12
	F-Score	98.43±0.79	98.47±0.79	94.46±1.91	94.86±1.34
	Kappa	97.76±0.85	98.96±0.22	94.05±1.50	94.87±2.19
	Training Time(s)	1845.40±49.75	1874.86±25.31	1841.50±18.75	523.00±11.13

**Table 4:** Classification accuracy assessment and computation cost with different models and pre-processing methods for cowpea.

		Cowpea			
Model		Pre-processing Dataset			
	Metrics	RAW	SDeri	SNV	LDA
Hybrid-CNN	OA	98.39±0.59	99.24±0.29	95.81±1.38	97.70±1.38
	F-Score	98.37±0.26	99.19±0.33	94.46±1.49	97.32±0.20
	Kappa	97.81±1.10	98.64±0.11	96.73±1.82	96.58±1.16
	Training Time(s)	971.37±8.01	950.60 ±4.23	964.01±1.85	403.97±16.72
3DCNN					
	OA	97.65±1.22	97.76±0.69	93.40±1.09	97.96±0.41
	F-Score	98.44±0.27	97.92±0.29	94.19±1.81	99.21±0.32
	Kappa	98.10±0.56	98.63±0.41	94.02±1.59	99.36±0.07
	Training Time(s)	2141.63±14.32	2190.13±27.17	2158.40±73.89	1017.50±79.53
2DCNN					
	OA	92.77±1.267	95.82±0.74	91.38±0.481	98.45±0.18
	F-Score	93.653±1.580	94.59±0.73	91.48±0.63	97.75±0.48
	Kappa	92.393±0.890	99.32±0.36	91.75±2.44	96.93±0.29
	Training Time(s)	774.00±1.85	851.03±32.63	872.30±15.99	398.57±41.27
HybridSN					
	OA	96.16±0.435	98.19±0.85	96.09±0.47	97.73±0.85
	F-Score	95.99±0.15	98.76±0.55	95.81±1.83	98.65±0.52
	Kappa	96.04±0.78	98.48±0.76	94.75±2.73	98.42±0.12
	Training Time(s)	1080.00±23.51	1049.53±18.32	1038.53±55.28	622.67± 23.05

The proposed model for both species exhibited good performance when trained with SNV- and SDeri-transformed datasets. Although the HybridSN and 3D CNN models had good performance when trained with SDeri datasets, their training time (caused by the high number of trainable parameters) made the implementation of this model a challenge. The proposed hybrid CNN model had reduced training parameters (< 200,000 trainable parameters), subsequently affecting the computational processing time. The SDeri-trained hybrid CNN model had a processing time reduced by over 1000 s as compared to the 3DCNN-SDeri model, which had a 2141.63 processing time for cowpea. For quinoa, the SDeri-hybrid CNN model had about 54.16% reduced training time when compared to the 3D CNN model (trained with a raw dataset, which had the highest training time of over 2000 s). For both species, the HybridSN-based models had a significantly high training time irrespective of the training dataset.

### 3.3 Effective wavelength selection

**Figure 7:**
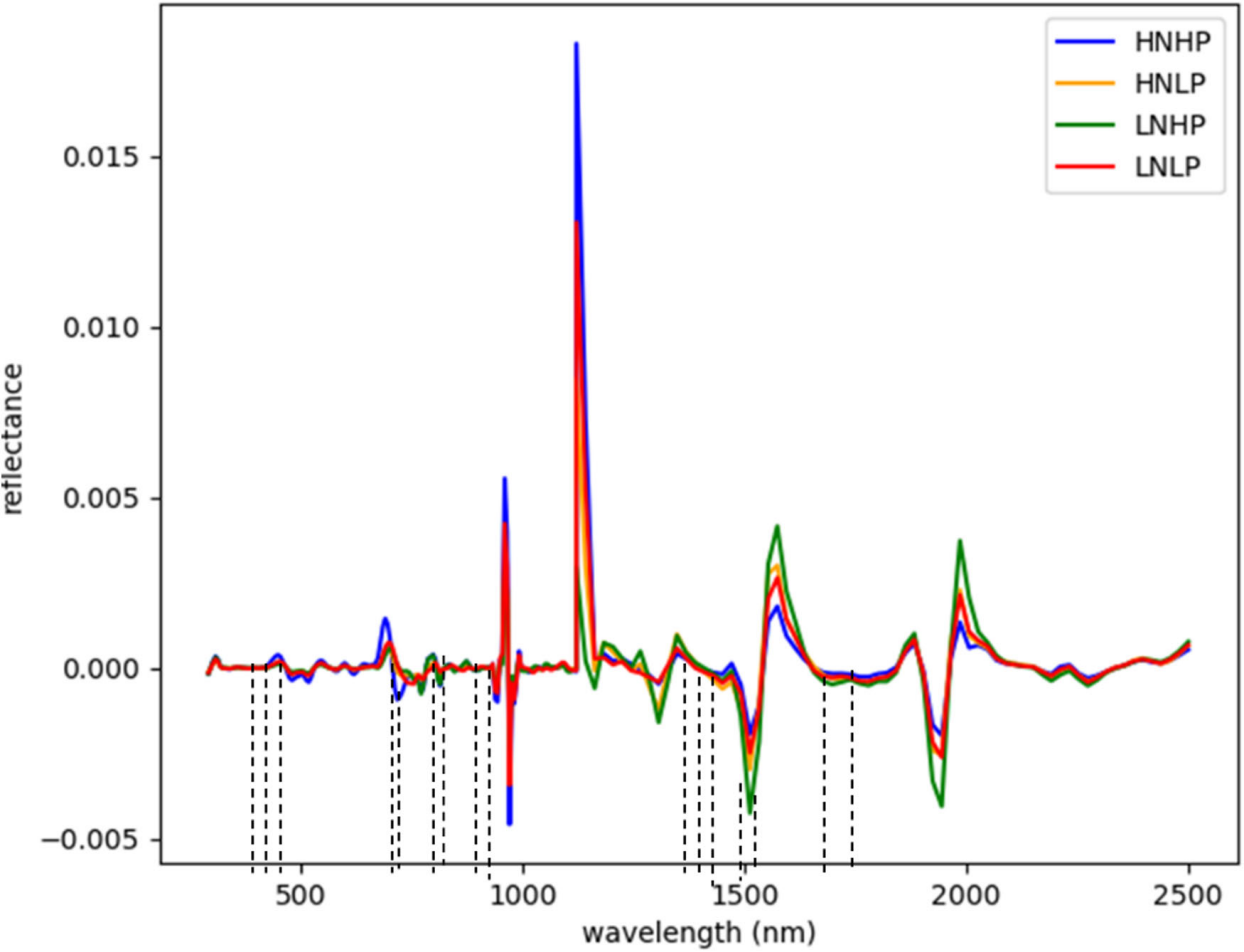
Selected wavebands using CFS method for cowpea; the short dash lines indicate selected band areas.

Although the proposed model with the SDeri dataset produced the best performance in classifying plant nutrient status, the high dimensionality of the hypercube posed computational challenges. Hence, effective wavelengths that accurately distinguish between the N and P levels were selected and used to retrain the models. From **Figure 7**, the selected wavelengths for cowpea were localized in the blue, red-edge, near-infrared, and short-wave infrared regions (having the highest number of selected wavelengths). Sixteen (16) wavebands were selected as the most sensitive wavelengths, with three selected from the blue regions (411, 431, and 455 nm). Moreover, two were selected from the red-edge regions (683 and 691 nm), four from the near-infrared regions (791, 871, 919, and 923 nm), and seven from the SWIR regions (1438, 1450, 1461, 1485, 1508, 1602, and 1660 nm).

**Figure 8:**
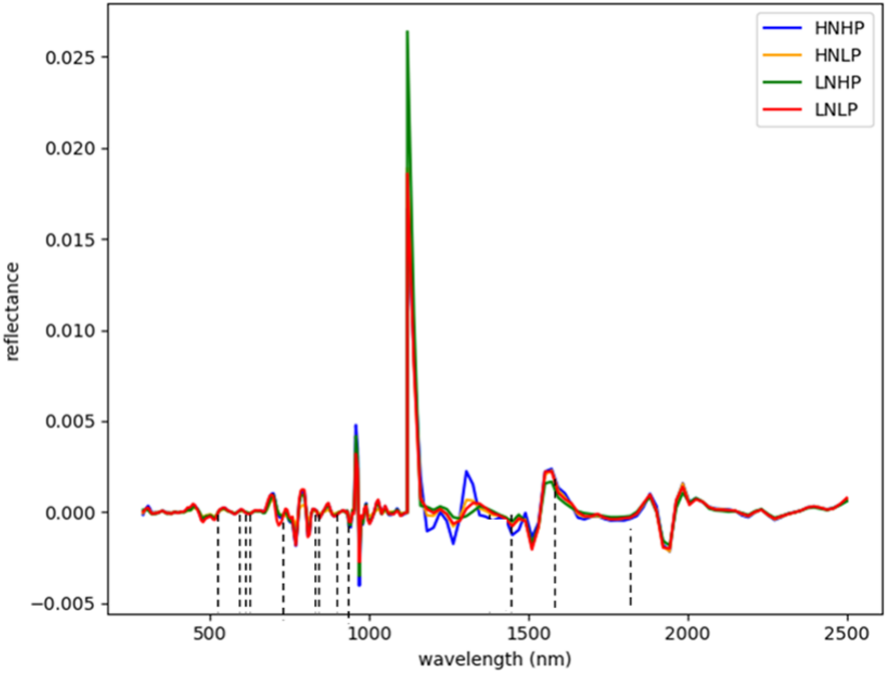
Selected wavebands using CFS method for quinoa; the short dash lines indicate selected band areas.

In quinoa, the wavelengths were selected within the blue, near-infrared, and SWIR regions. From **Figure 8**, twelve (12) wavebands were selected. These include four from the blue region (571, 575, 583, and 599 nm), one from the red spectrum (607 nm), four the near-infrared regions (723, 731, 924, and 967 nm), and three from the SWIR regions (1680 nm and 17520 nm). The differences in the selected wavebands could be due to the variability in the plant structure and the changes in the nutrient composition of the plants.

### 3.4 Results of discrimination analysis at different growth stages

After the waveband’s selection, the full plant (with full spatial resolution and selected spectral wavelength) in the second derivative format was used as a dataset to retrain the different models to predict the plants’ N and P statuses at different growth stages. Datasets were generated using the same approach as that in subsection 2.4.2. The training datasets were augmented to artificially enlarge the number of training images using rotation and flipping methods.

**Tables 5** and **6** show the performance of the models when trained with the selected wavelengths for cowpea and quinoa, respectively. All the models performed well (with 79-100% accuracy on the test data) irrespective of the growth stage under consideration. This performance was better than that found in Siedliska et al. (2021), where a 40-100% classification accuracy rate was achieved for predicting different P levels of three plant species at five growth stages. Comparing the four models, the proposed hybrid model had the best performance, attaining above 94% and 97% test accuracy across the selected growth stages for cowpea and quinoa, respectively. The 2DCNN model had the highest misclassification, with 84.08% in cowpea at growth stage IV and 79.23% in quinoa at growth stage II in the test dataset. For each species, there were high classification errors at the early growth stages, especially in the LNHP and HNLP plants. However, as the plants developed, there were clear distinctions between the spectral signatures of the different treatments, which resulted in an increase in the classification accuracy.

**Table 5:** Model performance on selected wavebands for classification of cowpea N and P levels at five growth stages.

	Plant	Cowpea					
		Training dataset (%)			Test dataset (%)		
Growth stage	Model	Accuracy	F-Score	Kappa Score	Accuracy	F-Score	Kappa Score
I	Hybrid CNN	99.12±0.10	99.61±0.08	99.35±0.27	98.51±0.25	98.88±0.25	98.44±0.31
	3D-CNN	96.21±0.41	96.08±0.21	96.02±0.13	93.27±0.66	93.12±0.76	93.04±0.0.44
	2D-CNN	93.25±0.14	93.21±0.56	93.17±0.25	89.21±0.17	88.71±0.17	89.21±0.37
	HybridSN	98.43±0.21	98.02±0.35	98.44±0.13	95.27±0.89	96.05±0.73	96.68±0.11
II	Hybrid CNN	99.26±0.50	99.05±0.25	99.08±0.21	99.48±0.56	99.25±0.33	99.51±0.62
	3D-CNN	97.15±0.10	97.22±0.16	97.21±0.53	94.11±0.06	94.41±0.25	94.05±0.81
	2D-CNN	95.00±0.13	95.16±0.12	95.33±0.26	91.13±0.26	91.22±0.17	91.05±0.11
	HybridSN	99.15±0.33	99.34±0.49	99.04±0.54	98.22±0.71	97.15±0.05	97.01±0.24
III	Hybrid CNN	99.54±0.24	99.42±0.11	99.63±0.12	98.68±0.22	98.41±0.34	98.22±0.21
	3D-CNN	97.41±0.15	97.51±0.24	97.25±0.84	93.74±0.41	93.51±0.25	93.41±0.45
	2D-CNN	96.28±0.64	96.12±0.85	99.18±0.15	92.19±0.37	91.87±0.14	91.59±0.26
	HybridSN	99.89±0.27	99.22±0.13	99.45±0.13	97.44±0.71	97.42±0.44	97.29±0.44
IV	Hybrid CNN	99.73±0.40	99.64±0.27	99.11±0.05	98.92±0.45	98.59±0.24	98.64±0.23
	3D-CNN	98.24±0.19	99.26±0.16	99.82±0.14	95.23±0.67	92.25±0.53	95.22±0.31
	2D-CNN	98.18±0.25	98.13±0.29	98.32±022	84.08±0.81	84.22±58	84.55±0.67
	HybridSN	98.51±0.22	98.17±0.31	98.25±0.35	86.44±0.7	86.02±0.51	86.58±0.74
V	Hybrid CNN	99.03±0.12	98.14±0.32	98.07±0.45	98.35±0.22	97.18±0.22	97.34±0.25
	3D-CNN	95.95±0.29	98.58±0.44	98.61±0.52	88.42±0.21	88.64±0.25	88.21±0.67
	2D-CNN	90.28±0.41	90.44±0.25	90.11±0.23	85.22±0.19	85.23±0.58	85.44±0.10
	HybridSN	98.66±0.44	96.35±0.28	96.46±0.15	92.42±0.16	92.25±0.44	92.41±0.31

**Table 6:** Model performance on selected wavebands for classification of quinoa N and P levels at five growth stages.

	Plant	Quinoa					
		Training dataset (%)			Test dataset (%)		
Growth stage	Model	Accuracy	F-Score	Kappa Score	Accuracy	F-Score	Kappa Score
I	Hybrid CNN	99.12±0.26	99.03±0.51	98.97±0.54	95.34±0.21	94.21±0.44	94.51±0.52
	3D-CNN	96.55±0.11	96.65±0.15	96.02±0.45	90.31±0.12	90.32±0.22	90.45±0.31
	2D-CNN	92.38±0.50	91.43±0.55	91.25±0.43	85.81±0.65	86.24±0.24	84.21±0.56
	HybridSN	97.21±0.25	97.02±0.35	97.42±0.11	91.65±0.33	90.15±0.40	90.16±0.25
II	Hybrid CNN	99.08±0.22	99.32±0.20	99.01±0.21	97.21±0.32	97.44±0.14	96.15±0.22
	3D-CNN	94.22±0.35	94.39±0.37	98.44±0.33	85.44±0.15	85.48±0.34	85.07±0.45
	2D-CNN	97.01±0.18	96.56±0.25	96.89±0.61	79.23±0.26	78.14±0.29	79.11±0.37
	HybridSN	96.55±0.16	96.23±0.32	96.34±0.20	85.15±0.22	85.33±0.32	85.01±0.43
III	Hybrid CNN	99.58±0.28	99.04±0.05	99.63±0.05	98.55±0.44	98.13±0.22	97.31±0.32
	3D-CNN	98.11±0.31	97.89±0.33	98.03±0.23	92.14±0.42	92.27±0.41	92.18±0.37
	2D-CNN	97.25±0.28	97.12±0.47	97.18±0.28	86.23±0.34	86.27±0.37	85.89±0.2
	HybridSN	96.89±0.12	96.63±0.45	96.55±0.21	85.41±0.29	85.25±0.22	85.09±0.29
IV	Hybrid CNN	99.29±0.44	99.18±0.51	99.23±0.25	98.29±0.29	98.80±0.27	97.74±±01.8
	3D-CNN	98.34±0.35	98.22±0.53	98.42±0.31	90.43±0.11	90.55±±0.23	90.3±0.36
	2D-CNN	96.81±0.36	96.43±0.25	96.32±0.14	84.18±0.45	82.33±0.15	82.45±0.22
	HybridSN	96.45±0.42	96.17±0.28	96.44±0.28	80.34±0.31	80.22±0.44	80.45±0.43
V	Hybrid CNN	97.72±0.37	97.54±0.14	97.28±0.25	95.18±0.26	95.23±0.58	95.27±0.12
	3D-CNN	96.05±0.22	95.78±0.33	96.11±0.37	89.32±0.12	89.53±0.24	89.11±0.31
	2D-CNN	95.44±0.12	95.14±0.16	9.31±0.42	82.15±0.41	82.43±0.15	82.18±0.36
	HybridSN	96.11±0.58	96.52±0.49	96.34±0.44	92.77±0.55	80.05±0.33	81.26±0.23

**Table 7** is the summary of the confusion matrix for the proposed model, which explains the accuracy and misclassification of the individual classes for cowpea and quinoa. For both species, the model accurately classified HNHP treatment across all the five growth stages. The model had difficulties in classifying some variants of the treatments, especially those with low phosphorus. In cowpea, HNLP (5% error) and LNHP (> 10% error) were misclassified at growth stage I. Moving to growth stage II, although a similar trend occurred, there was an improvement in the classification accuracy of LNHP (3.64% increment). In the subsequent growth stages, the model improved its performance, with > 95% at growth stages III, IV, and V. In quinoa, there was a 100% accuracy in the HNHP and HNLP classification. However, the model had lower performance in correctly classifying the LNHP treatment 92.82%. Moving across the growth spectrum, the model performance increased, achieving above 98% classification for HNHP and HNLP at growth stages IV and V.

**Table 7:** Summary of confusion matrices created for the proposed model for nutrient status identification at five stages of plant growth.

Plant	Treatment	Growth Stages				
		I	II	III	IV	V
Cowpea	HNHP	98.32	99.57	99.73	99.41	98.92
	HNLP	95.00	94.00	97.32	98.52	96.06
	LNHP	86.84	90.50	96.30	98.50	95.31
	LNLP	96.21	98.00	99.40	99.15	98.23
Quinoa	HNHP	97.15	99.41	99.23	99.10	98.65
	HNLP	90.22	95.12	98.01	99.27	96.14
	LNHP	92.82	97.21	95.13	99.27	98.88
	LNLP	98.52	96.75	98.31	98.58	98.25

## 4 Discussion

This study aimed to develop a deep learning-based HSI pipeline for classifying the N and P status in cowpea and quinoa at different growth stages. This was achieved by developing a hybrid 3D-2D CNN model to automatically learn and evaluate the spectral and spatial characteristics of the canopy components of the species. Previous studies have utilized 1D-, 2D-, and 3D-CNN models for hyperspectral image analysis. However, these models have limitations in terms of their feature extraction methods, which impact their performance. While 1D CNN extracts only spectral information and 2D CNN deals with spatial information, 3D-CNN extracts both spectral and spatial information simultaneously. Nonetheless, the complexity introduced by 3D CNN modeling can adversely affect its performance and output. Therefore, it is prudent to develop a model that combines 3D- and 2D-CNN architectures to learn features and patterns from spectral hypercube data while decreasing the computational complexities and processing time.

In this study, patches of hypercube (15 X 15 X 223) were extracted from the plant canopy with different N and P levels and used as an input to train the proposed model. Four different spectral transformation techniques were experimented to select the one with the most discriminative features for classifying plant nutrient levels. The performances of the models were compared with standard 3D-CNN, 2D-CNN, and HybridSN models (Section 3.3). As shown in **Table 3** (section 3.2), the proposed method achieved an accuracy of over 94% irrespective of the pre-processing technique used. However, the models trained with the second derivative spectra (SDeri) outperformed the others. The derivative processing reduced the background signal and image artifacts, which subsequently improved the discriminating power of the dataset. The high performance of the SDeri-based models agrees with the findings of Siedliska et al. (2021) and Luo et al. (2019), who achieved similar results using derivative spectra. Additionally, although LDA achieved comparably low classification accuracy, it improved the interpretability of the spectra information by replacing the original variables with a group of discriminants while preserving their original information.

A greedy stepwise CFS technique was applied to select the most informative set of wavelengths across the various spectral bands. The selected wavebands were used to retrain the models to identify the plant nutrient status at different growth stages. The proposed method achieved the highest performance, with over 94% accuracy for both species. Although the HybridSN and 3DCNN models performed well comparably, the high training time limited their practical application. Practically, the selected wavebands can be used to develop a multispectral imaging system to predict plant nutrient status. It should also be noted that all the models experienced a decrease in accuracy when classifying nutrient status at the early growth stages, particularly for the LNHP and HNLP treatments. This decrease in accuracy can be attributed to the fact that the differences in canopy spectral properties were considerably minimal between the four treatments at the early growth stage. As a result, it became difficult for the models to accurately differentiate the nutrient statuses based on spectral information alone during these early growth stages. This is challenging because, practically, farmers and plant breeders are more interested in identifying plant nutrient status at the early growth stage for proper nutrient management. Hence, further studies are required on the dynamic response of crop canopy to subtle changes in N and P concentration at the early growth stages using hyperspectral data.

## 5 Conclusion

Hyperspectral data hold significant potential for monitoring nitrogen and phosphorus nutrition of quinoa and cowpea, enabling the provision of optimal conditions for development and growth. This study has proposed the use of hyperspectral imaging in tandem with a hybrid 3D-2D CNN model to identify the nutrient status of cowpea and quinoa at selected growth stages. The experiment results presented demonstrate the capability of the proposed model to accurately distinguish plant nitrogen and phosphorus levels, based on selected wavebands from the second-order derivative of the reflectance spectra. The use of separable convolutions in the 2D CNN block of the proposed model reduces the model’s complexity by reducing the number of trainable parameters. This subsequently reduces the processing time while enhancing learning efficiency, which is advantageous. These findings suggest that the proposed model could be integrated into a system for the non-invasive detection of nitrogen and phosphorus deficiencies in precision agriculture. Moreover, the success of the waveband selection process shows the potential of developing a multispectral sensor system equipped with the selected wavebands as a viable alternative to hyperspectral imaging for nutrient stress detection. These findings highlight the potential of the proposed model for the early detection and precise management of nutrient stress in cowpea and quinoa plants.

## Data availability statement

The raw data supporting the conclusions of this article will be made available by the authors, without undue reservation.

## Author contributions

Conceptualization, FO and DC; methodology, FO and PS-T; software, FO and PS-T; validation, MH, FM, and DS; formal analysis, FO; investigation, NV and LG; resources, MC and AR; data curation, FO and DC; writing—original draft preparation, FO; writing—review and editing, MH, NV, FM, PS-T, DS, and LG; visualization and supervision, MH and FM; project administration, MM; funding acquisition, MH. All authors contributed to the article and approved the submitted version.
