# Supplementary material for: Modeling the spatial-spectral characteristics of plants for nutrient status identification using hyperspectral data and deep learning methods
Source: Front Plant Sci. 2023 Oct 16;14:1209500. doi: 10.3389/fpls.2023.1209500 (PMC10613979; doi:10.3389/fpls.2023.1209500)
Supplement: Supplementary file 1 [file DataSheet_1.docx]

Supplementary Data Sheet


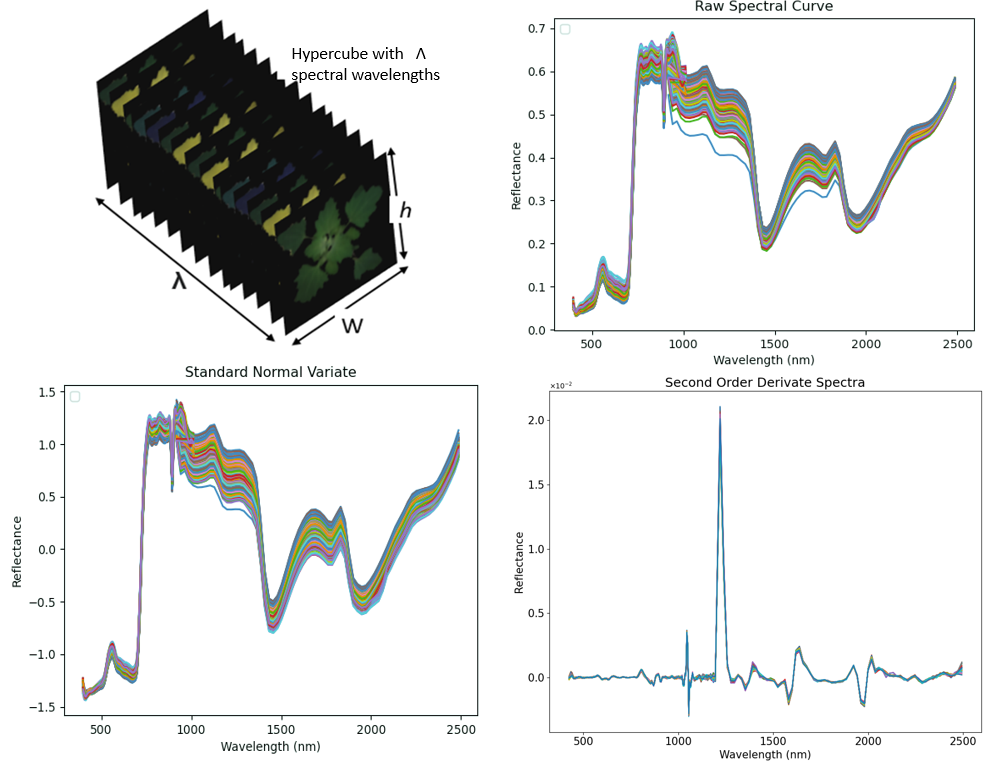


**Supplementary Figure 1**. Hyperspectral data extraction and different transformation techniques. (a) hypercube data of cowpea and quinoa (b) raw spectral curve (c) SNV-based curve (d) first derivative curve


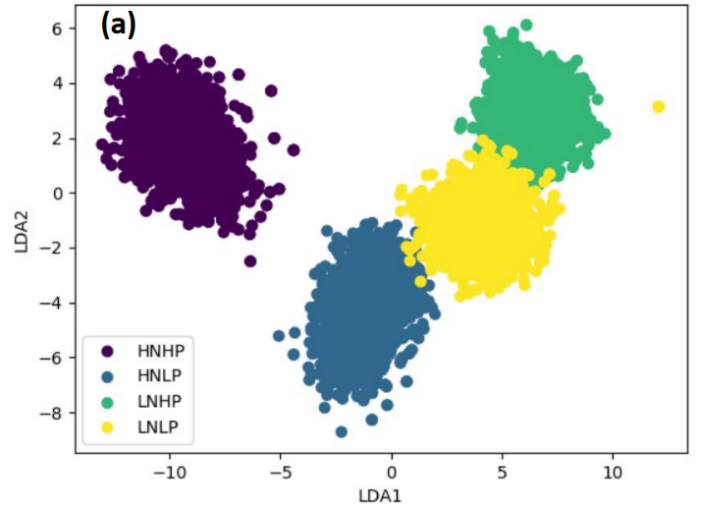

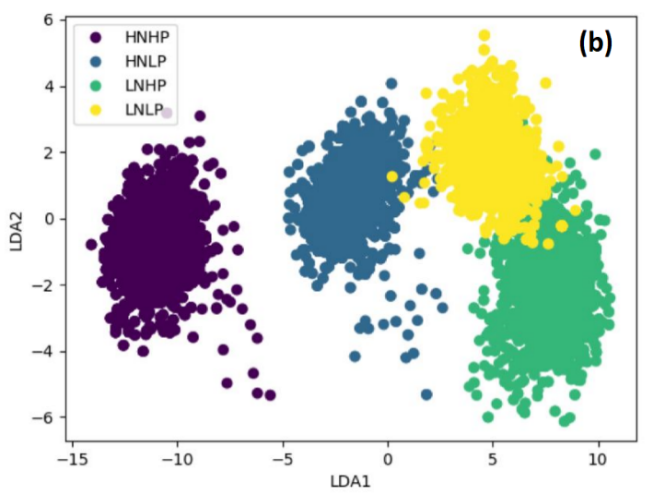


**Supplementary Figure 2**. Scores of scatter plots of the first and second LDA components (a) for cowpea and (b) for quinoa.


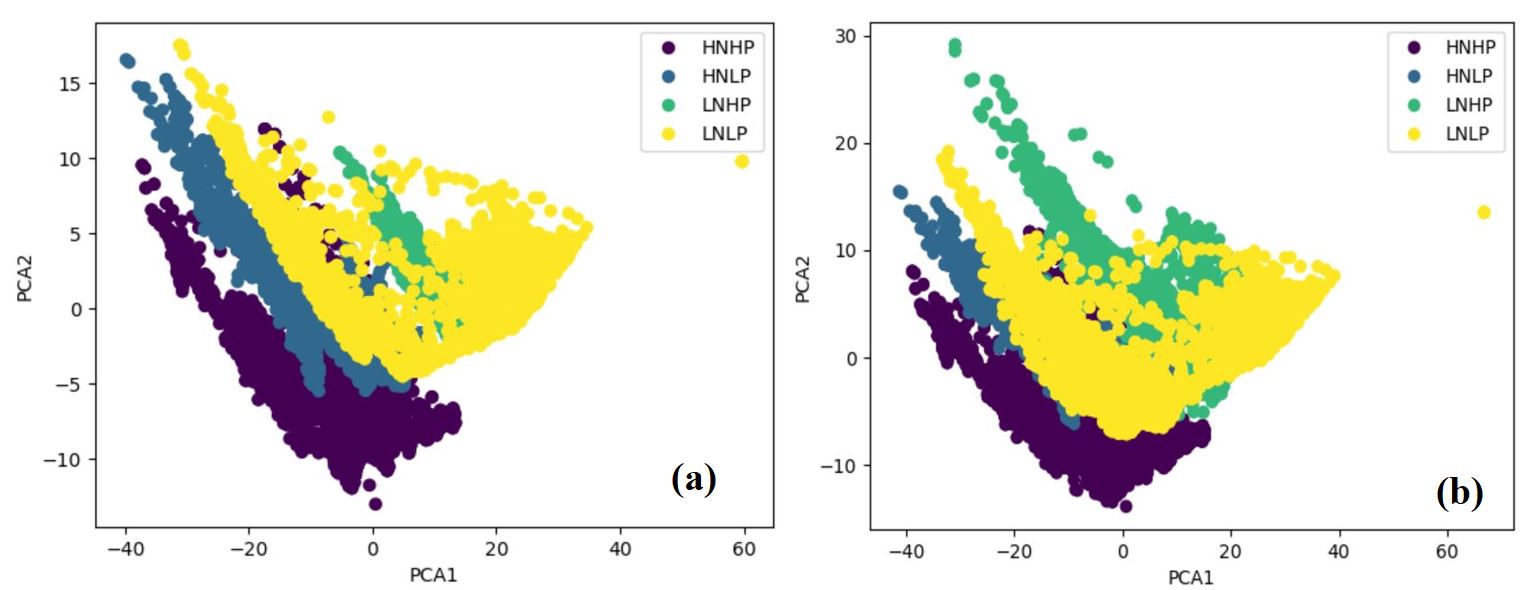


**Supplementary Figure 3**. Scores of scatter plots of the first and second PCA components (a) for cowpea and (b) for quinoa.


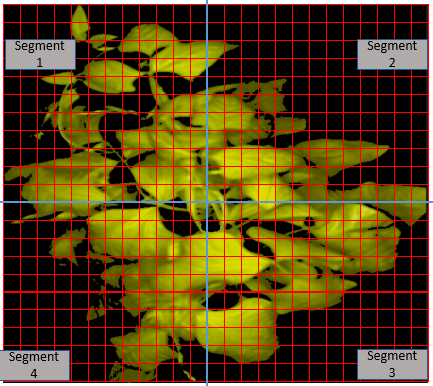

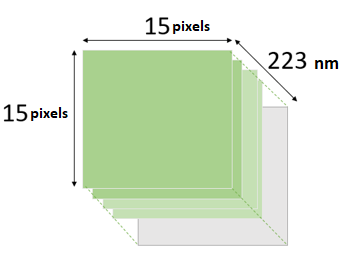


**Supplementary Figure 4**. Pseudo ‘RGB’ image showing patches (15 x15x223) for Cowpea.
