# Supplementary material for: Modeling the spatial-spectral characteristics of plants for nutrient status identification using hyperspectral data and deep learning methods
Source: Front Plant Sci. 2023 Oct 16;14:1209500. doi: 10.3389/fpls.2023.1209500 (PMC10613979; doi:10.3389/fpls.2023.1209500)
Supplement: Supplementary file 2 [file DataSheet_2.docx]

|  | Quinoa | |  | Cowpea |  |
| --- | --- | --- | --- | --- | --- |
| Growth Stage | DAT | Description | DAT | Description |  |
| I | 19 | Nine pair of leaves visible | 13 | 3 leaves (with stipules) unfolded, or 3 tendrils developed | |
| II | 29 | Nine side shoots visible | 30 | Beginning of stem elongation | |
| III | 36 | Stem elongation | 51 | First flower buds visible outside leaves | |
| IV | 50 | Inflorescence present but still enclosed by leaves | 60 | First flowers open (sporadically within the population) | |
| V | 69 | Complete anthesis: main inflorescence flowers with senesced anthers | 72 | 20% of pods have reached typical length | |

**Supplementary Table 1:** Selected growth stages of quinoa and cowpea based on the BBCH system.

| Layer No. | Layer Type | Layer details | Activation |
| --- | --- | --- | --- |
| 1 | Conv3D | filters = 8, kernel (3 × 3 × 3), stride = 1 | ReLU |
| 2 | Conv3D | filters = 16, kernel (3 × 3 × 5), stride = 1 | ReLU |
| 3 | Conv3D | filters = 32, kernel = 3 × 3 × 3, stride = 1 | ReLU |
| 4 | Reshape | Reshape 3D to 2D | - |
| 5 | Conv2D | filters = 64, kernel (3 × 3), stride = 1 | ReLU |
| 6 | Flatten | - | - |
| 7 | Dense | No. of Units = 256, dropout = 0.4 | ReLU |
| 8 | Dense | No. of Units = 128, dropout = 0.4 | ReLU |
| 9 | Dense | Output units = 4 (number of classes) | SoftMax |

**Supplementary Table 2:** Detailed architecture of HybridSN model

**Supplementary Table 3.** Detailed architecture of 3D CNN model for classification of plant nutrient status

| **Layer No.** | **Layer Type** | **Layer details** | **Activation** |
| --- | --- | --- | --- |
| 1 | Conv3D | filters (8), kernel (3 × 3 × 7), stride (1) | ReLU |
| 2 | Conv3D | filters (16), kernel (3 × 3 × 5), stride (1) | ReLU |
| 3 | Batch Normalization | 256 | - |
| 4 | Max Pooling | Pool size (2, 2, 2) | - |
| 5 | Conv3D | filters (32), kernel (3 × 3 × 3) stride (1) | ReLU |
| 6 | Conv3D | filters (64), kernel (3 × 3 × 7) stride (1) | ReLU |
| 7 | Batch Normalization | 128 | - |
|  | Max Pooling | Pool size (2, 2, 2) | - |
| 8 | Flatten | 516 | - |
| 9 | Dense | Units (256), dropout (0.4) | ReLU |
| 10 | Dense | Units (128), dropout (0.4) | ReLU |
| 11 | Dense | Output units (4) | SoftMax |

**Supplementary Table 4.** Architectural details of 2D-CNN model for classification of plant nutrient status

| **Layer No.** | **Layer Type** | **Layer details** | **Activation** |
| --- | --- | --- | --- |
| 1 | Conv2D | Filters (32), kernel (3 × 3) | ReLU |
| 2 | Max Pooling | Pool size (2, 2) | - |
| 3 | Conv2D | filters = 64, kernel = 3 × 3 | ReLU |
| 4 | Max Pooling | Pool size (2, 2) | - |
| 6 | Flatten | (256) |  |
| 7 | Fully connected | No. of Units (128), dropout (0) | ReLU |
| 9 | Dense | Output units (4)- number of classes | SoftMax |
